# *Salmonella*-based Rodenticides and Public Health

**DOI:** 10.3201/eid1006.030790

**Published:** 2004-06

**Authors:** John A. Painter, Kåre Mølbak, Jacob Sonne-Hansen, Tim Barrett, Joy G. Wells, Robert V. Tauxe

**Affiliations:** * Centers for Disease Control and Prevention, Atlanta, Georgia, USA;; † Statens Serum Institut, Copenhagen, Denmark

**Keywords:** Salmonella Infections, Salmonella enterica serotype Enteritidis, Rodenticides, Public Health, Zoonoses, Electrophoresis, Gel, Pulsed-Field

## Abstract

Several countries still permit strains of *Salmonella enterica* serotype Enteritidis, a leading cause of gastrointestinal illness in humans, to be used in rat baits. To assess the human health risk associated with such rat bait, we first reviewed historic data on health hazards associated with Ratin, a rodenticide that was used in Europe until the early 1960s. Ratin caused outbreaks of human illness, including several deaths. We then compared *S*. Enteritidis isolated from a current commercial product, Biorat, with *S*. Enteritidis from Ratin and found that the strains were both phage type 6a. Based on the similarity of the strains, currently available *Salmonella*-based rodenticides likely are as great a threat to public health as past strains were. Health officials should be aware that the continued use of *Salmonella*-based rodenticides is a risk to public health and should take appropriate measures to prevent use in their jurisdictions.

*Salmonella enterica* serotypes Typhimurium and Enteritidis have been used as rodenticides since the late nineteenth century. This use was explored after *S*. Typhimurium was discovered during a lethal epizootic in a research mouse colony (*1**,**2*). Researchers soon realized that the strains of *S*. Typhimurium used as rodenticide were identical to strains causing "meat poisoning" and might cause disease among humans. Use of *S*. Typhimurium rodenticides was discontinued early in the twentieth century, but *S*. Enteritidis continued to be used as a rodenticide in the United Kingdom and Denmark until the early 1960s. In 1954 (*3*) and again in 1967 (*4*), the World Health Organization (WHO) recommended that *Salmonella*-based rodenticides not be used because they posed a hazard to human health.

In spite of these recommendations, *Salmonella*-based rodenticides are still produced and used in Central America, South America, and Asia (*2**,**5*). Biorat (Labiofam, Cuba), one *Salmonella*-based rodenticide that is currently used in several countries (*6**,**7*), is made by coating rice grains with a combination of *S*. Enteritidis and warfarin. Currently, the Biorat product label offers no warning regarding the risk for human salmonellosis. Indeed, product information indicates that this product contains a strain of *Salmonella* that is pathogenic to animals but not to humans (*7*).

In 1995, the Centers for Disease Control and Prevention (CDC) received a sample of Biorat that had been distributed in Nicaragua (*2*), and in July 2001, U.S. custom authorities seized a shipment of Biorat destined for distribution in the United States. These incidents prompted us to compare Biorat with Ratin, one of the major *Salmonella*-based rodenticides used before the early 1960s; in addition, we summarize the public health hazards of *Salmonella*-based rodenticides.

## Microbiologic Findings

We compared three isolates of *S*. Enteritidis recovered from *Salmonella*-based rodenticides. Two isolates (of the Biorat strain) were from Biorat samples collected in 1995 and 2001. The label from the Biorat product obtained in 2001 states that Biorat contains 1.25% "monopathogenic" *Salmonella* and 0.02% hydroxycoumarin. Pooled samples of the 1995 Biorat product yielded 1x10^8^ CFU *S*. Enteritidis per gram of Biorat granules, and the 2001 product yielded 200,000 CFU per gram. A third isolate, *S*. Enteritidis var. Danysz (of the Ratin strain) was recovered from Ratin by the Danish Veterinary Laboratory in the 1920s or early 1930s.

Isolates were serotyped and biochemically characterized (*8*), subtyped by pulsed-field gel electrophoresis (PFGE) with the restriction enzymes *Xba*I and *Bln*I (*9*), and phage-typed. Phage-typing was performed at the National Laboratory for Enteric Pathogens, National Microbiology Laboratory, Canadian Science Centre for Human and Animal Health.

**Figure 1:**
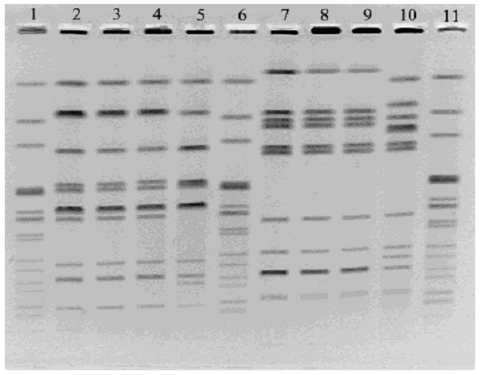
Pulsed-field gel electrophoresis of *Salmonella enterica* serotype Enteritidis isolates from Biorat and Ratin products using *Xba*I (lanes 2–5) and *Bln*I (lanes 7–10). Lanes 1, 6, and 11, molecular weight standard strain AM01144; lanes 2 and 7, Biorat isolate from 1998; lanes 3 and 8, Biorat isolate from 1995; lanes 4 and 9, Biorat isolate from 2001; lanes 5 and 10, Ratin isolate.

Both the Biorat strains and the Ratin strain were identified as *S*. Enteritidis, phage type (PT) 6a. The two Biorat strains were indistinguishable from each other by PFGE with the restriction enzymes *Xba*I and *Bln*I. PFGE patterns of the Ratin strain differed from those of the Biorat strain by three bands with *Xba*I and by five bands with *Bln*I (Figure 1).

Neither the Biorat strains nor the Ratin strain was shown to decarboxylate lysine. By contrast, four *S*. Enteritidis PT 6a isolates in the CDC culture collection from sources unrelated to Biorat or Ratin were positive for lysine decarboxylase. Threlfall et al. reported that the strain of *S*. Enteritidis PT 6a from Biorat is indistinguishable from those of the Ratin and Liverpool rodenticide strains by plasmid profile typing (*5*), as both strains contained plasmids of approximately 59, 4.0, and 3.0 MDa.

## Public Health Hazard

Since the mid 1980s, *S*. Enteritidis has caused a global pandemic of foodborne illness associated with eggs and poultry as a result of infection of the internal organs of chicken (*10*). Because of this pandemic, *S*. Enteritidis has become the most common serotype of *Salmonella* isolated from humans worldwide (*11*).

The *S*. Enteritidis strain found in Biorat is similar to the strain found in Ratin, a discontinued European product that caused human illness. Both strains were the same phage type, were indistinguishable by plasmid profile typing, and were different from 97% of salmonellae (*8*) in that they did not decarboxylate lysine. Although differences were noted between PFGE patterns of the Biorat strain and the Ratin strain, the similarities suggest that they may have originated from a common strain. Because the strains are similar and no evidence shows that the Biorat strain has decreased virulence, the Biorat strain is likely as pathogenic to humans as the Ratin strain.

In a retrospective study in Denmark from 1926 through 1956, Martin Kristensen identified 122 patients infected with the Ratin strain (*12* and unpub. data), including 5 (4%) who died, 3 of whom were children. Twenty-two (18%) of 122 patients were reported to have eaten food items contaminated with Ratin, while 43 (35%) had handled the rodenticide. In 1956, Taylor (*13*) also reported several outbreaks of food poisoning associated with *S*. Enteritidis rodenticides and concluded that the use of bacterial rodenticides should be stopped.

Rodenticides containing salmonellae were evaluated during a plague outbreak in San Francisco in 1895 (*2*); they were found to have no definable impact on the rodent population, but they caused illness and death in humans who prepared and handled them. In 1921, Willfuhr and Wendtland (*14*) reported several outbreaks of human *Salmonella* infections from rodenticides. In one of these outbreaks, Russian prisoners of war who ate a large number of Ratin potato baits became ill, and two died. In another outbreak in 1918, two persons died and approximately 35 became ill after eating a cake that had been intentionally contaminated with Ratin (*14**,**15*). From 1920 through 1940, other outbreaks associated with *Salmonella*-based rodenticides were reported, and several of these outbreaks included deaths (*15**–**18*).

The Biorat product insert, as well as information available on the Internet (*6**,**7*), claims that the product is not harmful to humans and does not contaminate the environment. Recent newspaper articles have generated interest in using *Salmonella*-based rodenticides as an alternative to chemical rodenticides. Advocates for the use of Biorat claim, "[Biorat] has absolutely no secondary effects on other animals, on the environment, or on humans…. It contains a strain of *Salmonella* that only affects rats" (*19*).

*S*. Enteritidis causes severe diarrheal illness, which can be life-threatening, especially among children, the elderly, and immunocompromised persons. We have not identified any peer-reviewed, scientific data on the safety of *Salmonella*-based rodenticides, and to our knowledge, all strains of *S*. Enteritidis are capable of causing human illness. Noting the hazards of *Salmonella*-based rodenticides, many countries have banned their use, and WHO has repeatedly recommended against use of salmonellae in rodenticides.

**Figure 2:**
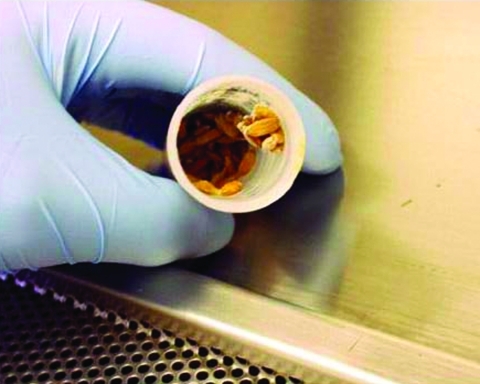
A sample of rodenticide that resembles grains of rice but contains pathogenic *Salmonella enterica* serotype Enteritidis.

Current concerns about bioterrorism suggest an additional public health threat posed by a commercially available strain of *S*. Enteritidis. *Salmonella*-based rodenticides have already been used intentionally to cause human illness (*14**,**15*); however, human illness may more commonly be caused by inadvertent exposure to *Salmonella*-based rodenticides. These rodenticides are generally mixed with grains to form baits (Figure 2). Biorat, for example, is made with whole rice and can easily be mistaken for food. Ingesting a few grams of bait, with at least 200,000 CFU per gram, could easily cause a severe case of salmonellosis.

To determine why *Salmonella*-based rodenticides are still used despite information about the public health hazards, we conducted a literature search with the keywords "*Salmonella*" and "rodenticide" (National Library of Medicine, http://www.ncbi.nlm.nih.gov). We found 10 articles, in addition to the recent public health publications discussed above (*2**,**5*); none addressed the public health hazards of *Salmonella*-based rodenticides. Many of the reference materials we used to prepare the present article were not written in English or were not retrievable from current electronic databases. The continued use of *Salmonella*-based rodenticides may likely be related to the fact that the content of important but dated scientific papers is unlikely to be known to current decision-makers.

*Salmonella*-based rodenticides may contain an approved rodenticide, such as warfarin, in concentrations high enough to kill rats, and the addition of *S*. Enteritidis has not been shown to increase the effectiveness of the poison (*2*). Extensive use of *Salmonella*-based rodenticides in the past may have increased the prevalence of *Salmonella* in rodents (*1*) and consequently increased the potential for human salmonellosis by transmission from rodents to food or food animals. Unfortunately, a misperception exists that some strains of *S*. Enteritidis are not pathogenic to humans. We recommend informing rodent-control authorities and the public that *S*. Enteritidis is a known human pathogen and that use of *Salmonella*-based rodenticides has had severe public health consequences. Effective and safe alternatives to *Salmonella*-based rodenticides are available worldwide.
